# Extrapolating Lessons from Targeted Protein Degradation to Other Proximity-Inducing Drugs

**DOI:** 10.1021/acschembio.4c00191

**Published:** 2024-09-12

**Authors:** Georg E. Winter

**Affiliations:** CeMM Research Center for Molecular Medicine of the Austrian Academy of Sciences, 1090 Vienna, Austria

## Abstract

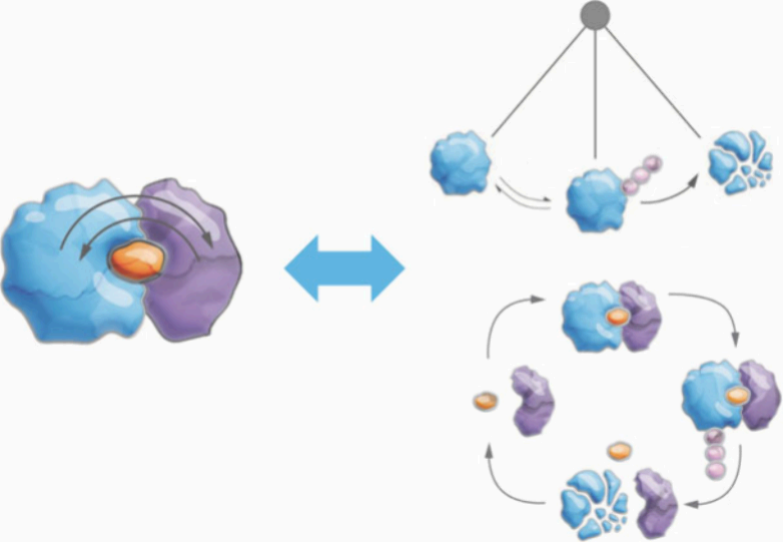

Targeted protein degradation (TPD) is an emerging pharmacologic strategy. It relies on small-molecule “degraders” that induce proximity of a component of an E3 ubiquitin ligase complex and a target protein to induce target ubiquitination and subsequent proteasomal degradation. Essentially, degraders thus expand the function of E3 ligases, allowing them to degrade proteins they would not recognize in the absence of the small molecule. Over the past decade, insights gained from identifying, designing, and characterizing various degraders have significantly enhanced our understanding of TPD mechanisms, precipitating in rational degrader discovery strategies. In this Account, I aim to explore how these insights can be extrapolated to anticipate both opportunities and challenges of utilizing the overarching concept of proximity-inducing pharmacology to manipulate other cellular circuits for the dissection of biological mechanisms and for therapeutic purposes.

## Introduction

Small molecules play a pivotal role as chemical probes, facilitating the dose- and time-dependent modulation of protein function to shed light on biological mechanisms. Throughout history, small molecules have also formed the cornerstone of many pharmaceutical treatments for human diseases. Despite the rising interest in biologics or cell-based therapies, most FDA-approved drugs are small-molecules.^1,2^ With few exceptions, both chemical probes and small-molecule drugs typically function by inhibiting the activity of isolated protein domains. This inhibitor-centric approach presents challenges for both drug discovery and basic research since only around 20% of human proteins are thought to be addressable with small-molecule inhibitors, even though covalent chemistry approaches promise to considerably expand this reach.^3−5^ Moreover, genetic loss of function (LOF) methods, including pooled CRISPR/Cas9 screens, predominantly alter protein levels rather than protein function. Consequently, replicating effects of genetic LOF through small-molecule inhibition can be challenging, particularly when the function relevant to a phenotype or disease is not directly influenced by the pharmacologically addressable domain.^6,7^ In essence, the inhibitor-centric paradigm restricts our ability to exploit small molecules for elucidating biological processes and confines the portion of the proteome deemed accessible for drug discovery efforts.

Targeted protein degradation (TPD) emerged as a promising pharmacological alternative poised to address the limitations of traditional small-molecule inhibitors. Mechanistically, TPD relies on small-molecule “degraders” that induce the proximity between an E3 ubiquitin ligase and a protein of interest (POI), leading to polyubiquitination of the POI and subsequent degradation via the ubiquitin-proteasome system (UPS). Analogous to genetic perturbations, degraders disrupt all functions associated with a target protein, bridging the gap between genetic LOF strategies and chemical inhibition in addressing mechanistic biological questions. Moreover, they offer the potential to expand the druggable proteome by rendering previously “undruggable” proteins susceptible to degradation.

**Figure 1:**
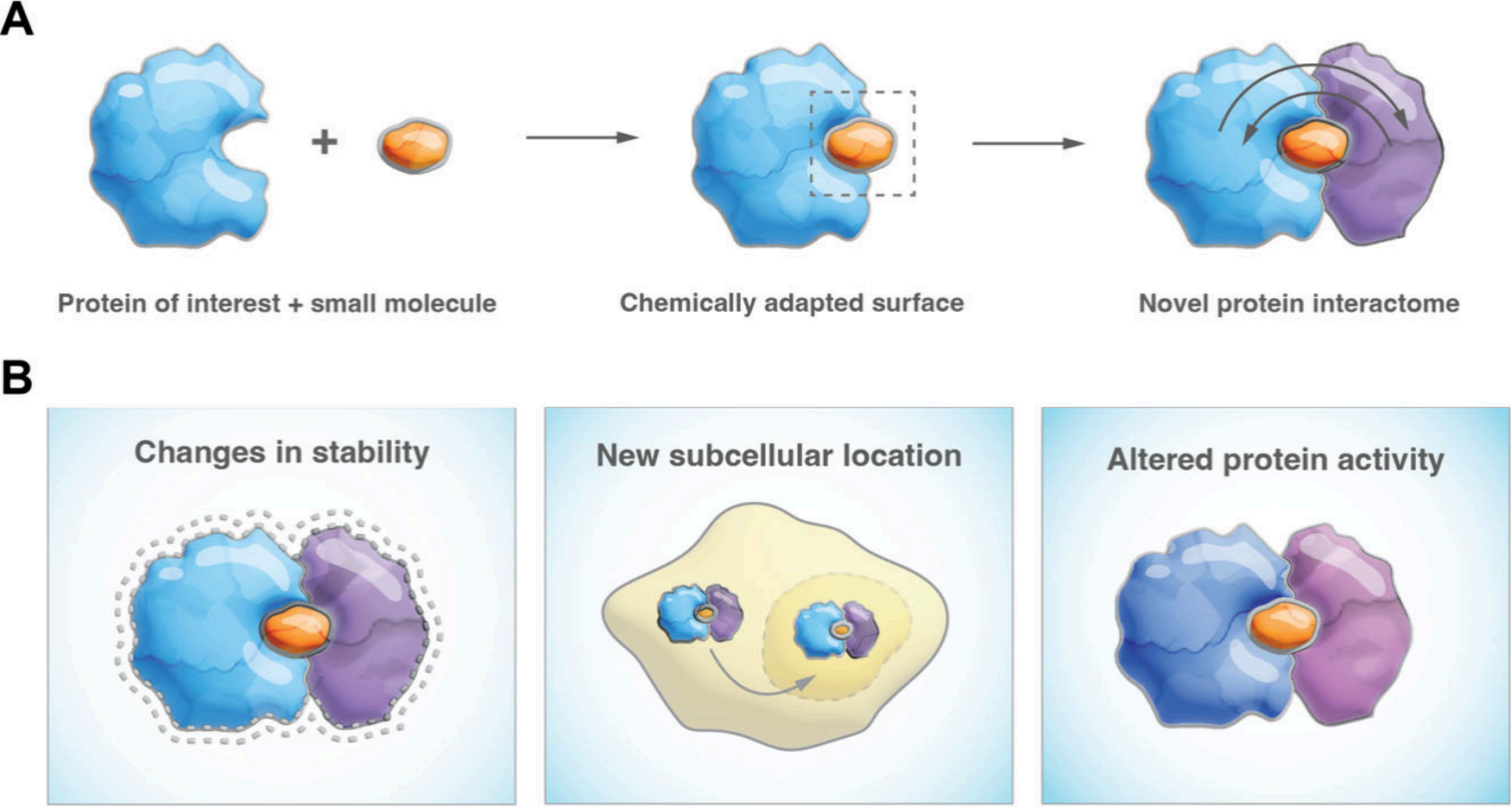
Proximity-inducing pharmacology via chemical neomorphs. (A) General logic of a chemical neomorph that binds to a protein of interest (POI), chemically rewires its surface properties to install or stabilize a novel protein–protein interaction. (B) High level description of how changes in the POI interactome can theoretically manifest in different POI fates and activities.

Following the thesis of the Accounts article series, I will here reflect on lessons we have learned in identifying, designing, and studying degraders to dissect biological mechanisms and to disrupt recalcitrant targets that were previously considered to be intractable for small molecule inhibitors. Specific attention will be directed toward highlighting intricacies of harnessing ubiquitin ligases. This should serve as a guiding principle to anticipate and evaluate opportunities, challenges, and potential impediments in broadening the scope of neomorphic, proximity-inducing pharmacology (PIP) to reprogram other cellular processes, such as signal transduction, protein localization, or transcriptional regulation (Figure 1). Due to our research interest, this perspective exclusively focuses on small-molecule approaches. Excellent reviews that cover antibody-based modalities and extracellular degradation strategies have recently been published.^8−11^

## Design, Identification, and Mechanistic Characterization of Degraders

### Designing and Characterizing PROTACs

**Figure 2:**
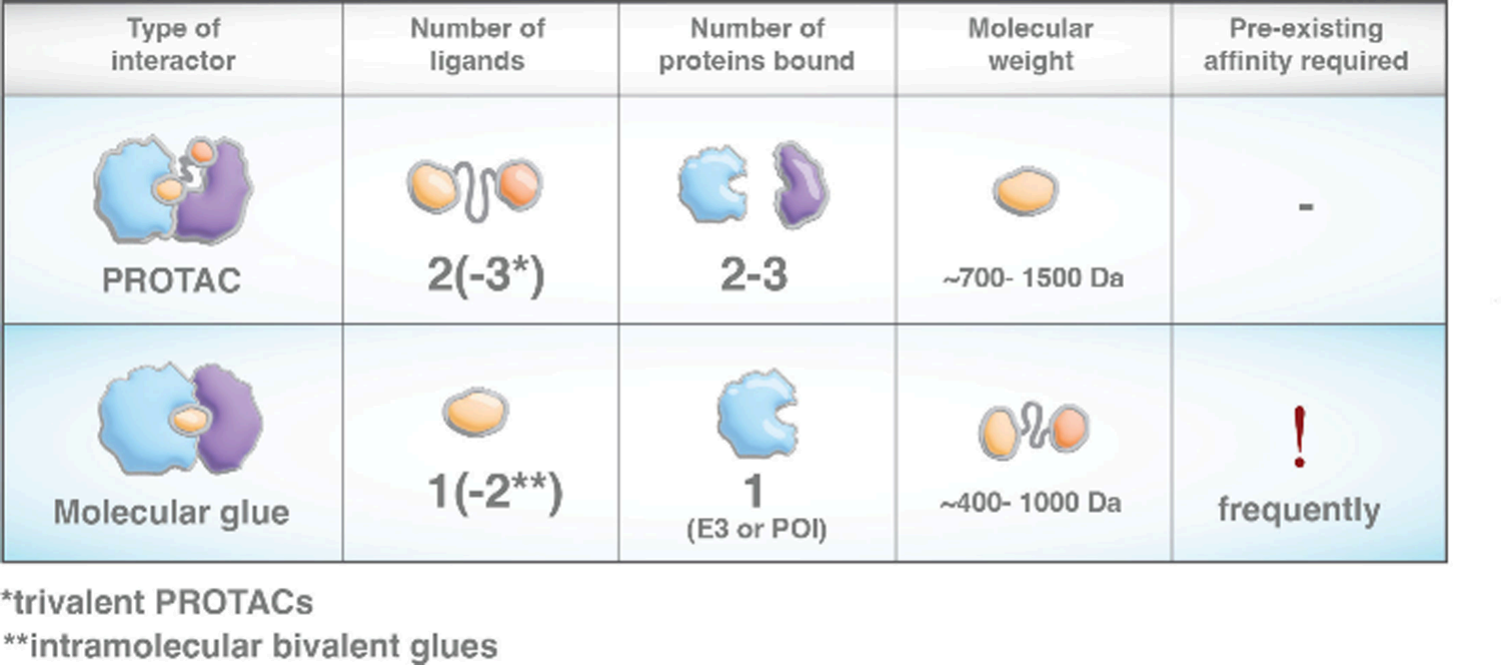
Two main classes of small-molecule degraders. Schematic comparison of key characteristics of heterobifunctional degraders (PROTACs, including trivalent PROTACs)^14^ that act in *trans* and molecular glue degraders (MGDs, including intramolecular bivalent glue degraders)^15^ that act in *cis*. MGDs typically only engage one protein: the POI or the E3/effector. Pre-existing affinity relates to affinities between POI and effector.

The general topic and history of TPD has been subject of various excellent reviews and will hence not be covered in depth here.^12,13^ Briefly, the field traditionally distinguishes between heterobifunctional degraders (known as “PROTACs” for Proteolysis Targeting Chimeras) and monovalent molecular glue degraders (MGDs). Broadly speaking, both types induce molecular proximity between a protein of interest (POI) and an E3 ubiquitin ligase, albeit through different mechanistic principles. PROTACs facilitate proximity by simultaneously engaging both the POI and the E3 ligase using defined ligands connected via a linker region. In contrast, MGDs typically only engage the ligase or the target, thereby creating a composite protein–drug interface that cooperatively stabilizes the interaction between both proteins (Figure 2). However, as our understanding of the precise mode of action of various degraders improves, the distinctions between these two categories become increasingly blurred. For example, many potent PROTACs also induce a cooperative ternary complex involving the E3 ligase, degrader, and POI, often due to a heavily rigidified linker element that lacks the flexibility inherent in initial PROTAC design. Additionally, MGD-like behavior can also be observed with heterobifunctional molecules (Figure 2 and outlined below).

Our interest in TPD was initially in heterobifunctional degraders. On the heels of the identification of the E3 ligase CRBN as the cellular target of thalidomide,^16^ we developed the first CRBN-based PROTACs that leverage drug-like E3 ligase binders, alongside other seminal studies focusing on the E3 ligase VHL.^17−19^ Compared to previous designs, our phthalimide-conjugated, CRBN-based degraders facilitated, for the first time, *in vivo* targeted protein degradation in mouse xenograft studies. Moreover, we could couple degrader treatment to proteomics profiling, revealing the selective on-target efficacy of those molecules.^17^ Intrigued by the selective degradation activity of prototypic PROTACs targeting BET family proteins, we delved further into the selectivity profiling of degraders, particularly focusing on ATP-competitive kinase inhibitors as targeting ligands. Due to the high conservation of the ATP-binding pocket among the more than 500 kinases encoded in the human genome, many kinase inhibitors bind to numerous kinases within a concentration range actionable for degraders (nanomolar to low micromolar). Consequently, these inhibitors present an ideal platform for investigating the parameters distinguishing binding from ensuing degradation. Contrary to our studies with BET bromodomain degraders, we and others observed that employing multitargeted kinase inhibitors as PROTAC targeting ligands often yields specific kinase degraders.^20^ For instance, we found that a PROTAC that leverages the multikinase inhibitor SNS-032 as targeting ligand functions as a highly specific degrader. Despite binding several kinases, the SNS-032-derived PROTAC (THAL-SNS-032) selectively degraded CDK9 on a proteome-wide scale.^20^ These observations provided compelling evidence that converting an inhibitor into a degrader can offer a means to engineer selectivity, a particularly formidable challenge in target protein families such as kinases, where high sequence homology of the active site complicates the design of truly selective inhibitors. Alongside other researchers in the field, we demonstrated that highly selective degraders can be engineered for kinases, for instance enabling the selective degradation of CDK6 over the closely related CDK4.^21−23^ Subsequent large-scale studies on kinases and HDACs support the notion that PROTACs frequently degrade only a subset of the targets that they are binding.^24,25^ Mechanistically, degradation selectivity often arises from the preferential architecture of a ternary complex. It is frequently explained by structural determinants outside the actual ligand binding site or via differential accessibility of lysine residues in the POI.^22,26−28^ Noteworthy, a selective degrader that has been derived from a multitargeted inhibitor might still elicit broad-spectrum inhibition of many of the bound kinases, which could cause unwanted side effects. To maximize the utility of such a degrader as a therapeutic agent (but also as a chemical probe), additional optimizations might be required to reduce promiscuous kinase inhibition and offset the anticipated drop in degradation efficiency by increasing catalytic turnover and cooperativity elicited by the degrader (see Figures 4 and 5).

However, to fully understand the selectivity profile of PROTACs, one needs to also be aware of inadvertent modulation of E3 ligase preference. For instance, we and others have shown that CRBN-based PROTACs may exhibit unanticipated off-target effects, such as targeting GSPT1, attributable to the molecular glue-like behavior of the employed CRBN-targeting ligand.^29,30^ Additional off-target neosubstrates with potential clinical implications are IKZF1/3 or SALL4. Of note, recent studies highlight that also VHL-targeting ligands could feature glue-like properties.^31^ However, the field has made significant strides in understanding chemical strategies to mitigate such glue-like behavior.^32^

On the shoulders of advances in academic research, translational efforts led by biotech and biopharmaceutical industries start to bear fruits. Presently, over 20 different PROTACs have entered human clinical investigation, with promising data emerging for programs targeting the estrogen receptor, BTK, IRAK4, and others.^12^ While most of these clinically pursued targets are *per se* not undrugged or considered to be “undruggable”, PROTACs are nevertheless expected to deliver a differentiated therapeutic benefit since they can, for instance, address mutations that convey resistance against inhibitors.^33^ Moreover, based on their catalytic mode of action, degraders can also provide a deeper perturbation of the disease biology associated with the addressed target and, due to the prolonged effect duration, enable less frequent dosing compared to occupancy-driven inhibitors (see Figure 5).

As PROTACs progress clinically, we will also learn to understand which resistance mechanisms might come into play and how often the co-opted E3 ligase will be affected. We and others have explored these issues in preclinical resistance models, including large-scale CRISPR LOF screens or deep mutational scanning (DMS).^34−38^ Collectively, these data suggest that the strongest selective pressure will be on inactivating the E3 ligase or abrogating degrader binding to the E3. However, further studies are required to determine which of these findings will be replicated in patients. In support of an outsized contribution of the E3, recent studies indicate that approximately one-third of all patients relapsing from the CRBN-based MGDs lenalidomide or pomalidomide feature mutations in CRBN or associated regulators.^39^

### Identifying and Characterizing Molecular Glue Degraders

Historically, the “molecular glue degrader” terminology was coined to describe the mode of action of the plant hormone auxin.^40,41^ The ensuing identification of thalidomide and the closely related lenalidomide and pomalidomide (“IMiDs”) as MGDs revealed this mechanism of action as a pharmacologically actionable modality.^42,43^ In essence, IMiDs bind to CRBN, leading to the recruitment, ubiquitination, and degradation of the C2H2 zinc finger transcription factors IKZF1 and IKZF3. Thereby, IMiDs partially mimic the naturally occurring CRBN degron represented by C-terminal cyclic imides.^44^ While initially perceived as a unique “black swan” event, multiple lines of evidence suggested that MGDs are likely more widespread. This included, among others, the expansion of the target space of CRBN-based glue degrader and the identification of aryl sulfonamides as DCAF15-dependent MGDs.^45−47^ Moreover, the protein interfaces involved in the gluing process appeared often nonconserved, arguing against an evolutionary optimized ternary assembly.^48^ Additionally, orthogonal evidence from mutational studies indicates that protein surfaces have evolved at the cusp of supramolecular self-assembly, suggesting that even minor modifications to protein surfaces may suffice to induce or stabilize protein–protein interactions (PPIs).^49^

Encouraged by these observations and advancements, our team, along with others, embarked on developing strategies to facilitate the identification and in-depth mechanistic characterization of MGDs.^34^ Genome-wide CRISPR/Cas9 loss-of-function (LOF) screens, conducted by us and other groups, enabled mapping of genes essential for the action of PROTACs and MGDs.^34,35^ Among these genes, *UBE2M* stood out as its disruption conferred universal resistance to all tested degraders. UBE2M plays a crucial role in adding the post-translational modification NEDD8 to all cullin backbones except cullin 5, a process known as “neddylation”, which is essential for the activity of the corresponding cullin-RING E3 ligase (CRL) complexes.^50^ Hence, we surmised that *UBE2M* mutations should induce a widespread loss in the activity of more than 200 CRLs. We could validate this hypothesis in an ensuing study, which prompted us to test if comparative chemical profiling in cell lines that are either *wildtype* or mutant for *UBE2M* would enable us to find novel degraders.^51^ Encouragingly, testing around two thousand small molecules in both genetic backgrounds led us to identify a structurally novel compound that induces the degradation of RBM39 by recruiting it to the DCAF15 ligase. Motivated by this finding, we mechanistically investigated another chemical series. Coupling expression proteomics with an array of functional genomics approaches, we could reveal a unique and unexpected degrader mechanism of action. In brief, we found that these compounds bind to the ATP-binding site of CDK12 and CDK13. Consequently, CDK12/13 get “glued” onto the DDB1 adapter protein of a CRL4B ligase, prompting the highly selective degradation of Cyclin K (CycK), the cyclin that is bound to and regulates the activity of CDK12 and CDK13. Noteworthy, several independent studies have found additional compounds that function via this mechanism, thus highlighting that the CDK12/13:DDB1 interface is likely primed for induced proximity.^52,53^ A structural rationale for this widespread mechanism has been provided by a recent manuscript.^54^ In a separate initiative, we aimed to enhance the repertoire of tools available for the discovery of novel MGDs. Our objective was to develop an approach enabling the identification of MGDs tailored for a predefined CRL in a target-agnostic manner. To achieve this, we devised a strategy termed “ligase tracing.″ This method entails initiating the controlled autodegradation of a specific CRL lacking a substrate. Subsequently, MGDs are identified by screening for molecules capable of stabilizing the autodegrading ligase through chemical recruitment of a (neo-) substrate. In a proof-of-concept screen, this led to the discovery of dRRM-1, a novel DCAF15-dependent MGD of the splicing factors RBM23 and RBM39.^55^ Another strategy we explored involved the identification of MGDs through morphological profiling. In brief, we developed a direct to biology (D2B) approach that enabled us to synthesize a bespoke library of CRBN-binders and directly assay them in a set of isogenic cell lines that feature different levels of expression of CRBN. Since most other cell-based MGD discovery approaches have focused on a viability-based readout, we here decided to couple cellular treatment with morphological profiling via the cell painting assay.^56−58^ This led, among others, to the identification of a MGD selectively targeting GSPT2. Many other creative approaches to identify or characterize MGDs have been brought forward, leveraging both cellular as well as recombinant approaches. This includes cellular positive selection (“up assay”) screens to identify novel degraders of IKZF1, or recombinant high throughput screens coupled with structure-based optimization to restore an E3-substrate interaction that is abrogated by disease-relevant mutations, as exemplified by compounds gluing mutant β-TrCP and β-catenin.^59,60^ Moreover, recent work highlights how DNA-encoded library (DEL) selections can be leveraged to find cooperative MGDs, or how activity-based probes of CRL networks can reveal degrader mechanisms.^61−63^ Additional approaches for MGD discovery are covered in an excellent recent review.^64^

In addition to developing unbiased approaches for MGD discovery, novel strategies for degrader design can also emerge from detailed mechanistic investigations of serendipitously identified degraders, as demonstrated by the aforementioned work on IMiDs and aryl sulfonamides. This is also shown by an impressive mechanistic workup of the mode of action of the monovalent BCL6 degrader BI-3802 that functions by inducing specific and reversible BCL6 polymers, which are recognized and degraded via the SIAH1 E3 ligase complex.^65^ Key to these efforts are unbiased approaches such as time-controlled, FACS-based CRISPR screens, quantitative proteomics and deep mutational scanning approaches.^66−68^ These techniques enable a comprehensive understanding of the molecular mechanisms underpinning degrader activity and thereby facilitate the rational design of next-generation degraders with enhanced efficacy and specificity.

Recently, we have coupled such unbiased strategies to biochemical reconstitutions and structural studies to uncover the mechanism of action of a BRD4 degrader disclosed in the patent literature.^15^ Despite its traditional PROTAC-like structure and its ability to bind to the DCAF15 ligase in biochemical assays, we were surprised to discover that this compound exhibited functional independence from DCAF15. Through time-controlled, FACS-based CRISPR screens, we successfully identified and validated DCAF16 as the functionally required ligase. It is noteworthy that despite their nomenclature, DCAF16 and DCAF15 exhibit significant structural differences, as predicted by alphafold models.^69^ This unexpected finding was further compounded by the fact that the degrader did not exhibit binding to DCAF16 in recombinant assays. Interestingly, through various mutations, truncations, and domain-swaps, we could identify that sustained activity of the degrader depends on both N-terminal bromodomains of BRD4, a prerequisite that we have not encountered previously. Further insights emerged upon solving the cryo-EM structure of the ternary assembly, revealing that the bifunctional degrader engaged both BRD4 bromodomains *in cis*. This nucleated a surface conformation that interacts with DCAF16 through various PPIs and protein–drug interactions. These findings led us to designate the compound as an intramolecular bivalent glue degrader (IBG1).^15^ Based on the established mode of action, we could synthesize analogs with improved cellular potency. Importantly, by extending our studies to other compounds, we identified IBG4, another intramolecular bivalent glue degrader of BRD4 that functions by co-opting the E3 ligase DCAF11. This demonstrated the extensibility of this approach across different E3 ligases. Our findings suggest intramolecular glue degraders as a differentiated degrader modality, where bivalent, intramolecular target engagement modulates protein surface composition to stabilize PPIs. Given that up to 80% of all human proteins possess more than one domain,^70^ we anticipate that this approach holds broad applicability and may offer advantages in terms of selectivity and benefit from avidity.

In summary, detailed mechanistic investigations of serendipitously identified degraders continue to serve as a source of innovation and complement dedicated discovery approaches. These studies rely on open-ended and unbiased cell-based methodologies to elucidate and comprehend the molecular mechanisms at a high level of granularity. This is essential for forming mechanistic hypotheses that can then be translated into reductionistic models for biochemical and structural validations and assays that can drive degrader SAR studies.

## Probing and Dissecting Biology with Small Molecules and Degraders

**Figure 3:**
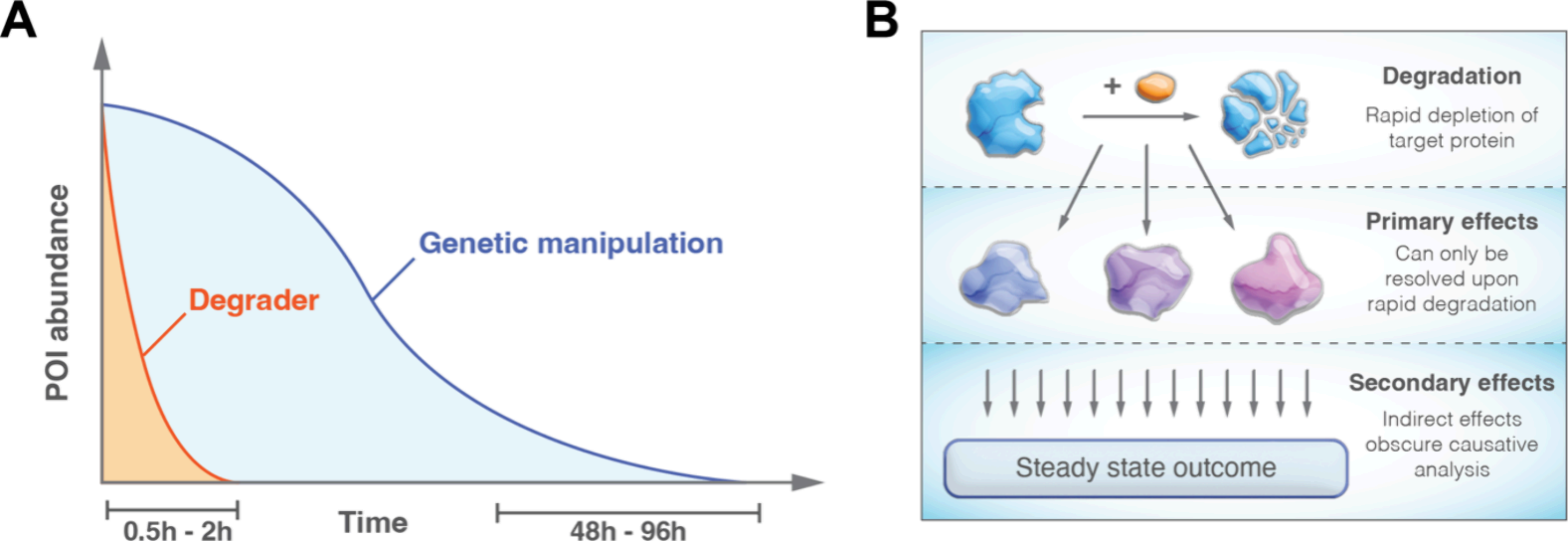
Degraders facilitate a mechanistic understanding. (A) Directly acting and genetically encoded small-molecule degraders typically operate within a few hours and thus at a different time scale than most genetic perturbations. (B) Through this higher kinetic resolution, degraders can help to identify primary mechanistic consequences and approximate causality in biological systems.

The ever-increasing resolution and depth that is provided by the application and integration of various -omics technologies continue to unravel novel layers of complexity in and spatial organization of biological systems. However, comparative steady-state analyses, for instance in knockout cell lines, can hamper our ability to separate cause from consequence.^71^ Focusing on inducible processes can circumvent this bottleneck. Another strategy to discern direct mechanisms from secondary consequences is to focus on biochemical reconstitutions. While such experiments allow well-controlled conditions, they are limited in terms of the complexity they can access. Small-molecule inhibitors and degraders that meet the criteria of chemical probes offer means to address and approximate causality also in biological systems (Figure 3).^71,72^

Toward that goal, several parameters are of critical importance. First, the kinetics of the biological process under investigation needs to be matched with kinetics of action of the compound and the experimental readout. This ensures that acute probing can have a measurable biochemical or phenotypic consequence. Moreover, selecting appropriate negative control compounds with physicochemical properties closely matched to the compound of interest, or designing genetic rescue experiments (such as introducing drug resistant/binding-deficient mutants), will help to confirm that the observed mechanisms are indeed on target.^73^ In recent years, the high kinetic resolution provided by directly acting small-molecule degraders or genetically encoded degron systems has been widely adopted and continues to garner attention.^74−77^ Among other applications, the field of transcription regulation has particularly benefitted from these systems.^71,78,79^ We have developed a particular interest in applying small-molecule degraders to understand transcription regulation and associated transcriptional addictions in various cancers. In most cases, we found that matching acute perturbations with fast-acting, nascent transcriptomics technologies to be most informative. This led us and others to identify the essential role of BRD4 in transcription elongation independent of pTEF-b recruitment.^80,81^ Acute degradation of BRD4 led to a complete disruption of transcription elongation and a global downregulation of mRNA levels followed by acute induction of apoptosis. These data offered a compelling hypothesis explaining why BRD4 degradation was much more cytotoxic compared to BRD4 bromodomain inhibition which prompts a more focused transcriptional response.^80^ Noteworthy, recent evidence points to an additional contribution of proteolytic peptides generated due to BET degradation in the induction of apoptosis via blocking Inhibitor of Apoptosis proteins.^82^ The extent of this contribution and generalizability to other degraders will be addressed in future research. The implementation of the degradation tag (“dTAG”) technology moreover enabled us to dissect the transcriptional addiction of acute leukemias on the elongation factor ENL or allowed us to uncover immediate consequences in disrupting human Mediator.^77,83^ Over the last couple of years, several orthogonal genetically encoded degron systems with unique advantages and limitations now offer a broad variety of experimental systems that investigators can choose and combine, as needed.^75,84,85^ Many additional studies from colleagues in the field have likewise illuminated our understanding of transcription regulation, chromatin structure or many other biological processes.^71^ En route to a better understanding of fundamental mechanisms, many of these studies have also contributed to preclinical target validation that can inform candidate properties in drug discovery campaigns.^86,87^ In conclusion, I am confident that the integration of pharmacologic degradation approaches with unbiased and rapid biological readouts will remain instrumental in advancing causal mechanistic studies. Exciting avenues lie ahead, particularly in approaches that facilitate the generation of chemically actionable alleles at scale, such as through pooled knock-in strategies.^88−90^ This advancement will enable combining acute perturbations of hundreds of proteins with single-cell technologies, thereby facilitating the elucidation of their effects on transcription, chromatin accessibility, or protein localization at scale.

## From Degraders to a General Concept of Chemical Neomorphs

The growing interest in PROTACs and MGDs across academic research and the pharmaceutical industry has motivated efforts to reprogram other biological circuits using chemical neomorphs. Of note, recent findings outline the fascinating possibility that small-molecule binding can activate an otherwise latent enzymatic function of the target protein without the need of an additional effector protein.^91^ At this point, we still need to learn how similarly acting molecules can be identified or rationally designed, and we also need to understand the potential scope of target proteins that this might apply for. Currently, efforts to expand the reach of chemical neomorphs primarily focus on proximity-inducing pharmacologic (PIP) strategies. Following the lead of PROTACs, most of these strategies aim to recruit effectors to target proteins to induce posttranslational modifications (PTMs), akin to how PROTAC-mediated E3 ligase recruitment prompts target protein ubiquitination. These approaches have been successfully implemented for targeted phosphorylation, dephosphorylation, acetylation, or glycosylation, among others. Rather than recapitulating the individual approaches, I would like to point the reader to a set of excellent recent reviews that cover the expanding landscape of proximity-inducing chemical strategies that modulate posttranslational modifications on target proteins.^92−94^

Instead, I would like to reflect on how the steep learning curve in the TPD field might help us to predict future trends and possible challenges in this space by emphasizing aspects of TPD that might be nontrivial to emulate for other PIP modalities. By and large, it is probably fair to assume that many of the chemical degrader design principles will also apply to other PIP modalities, including an outsized contribution of linker design and linker architecture as well as general medicinal chemistry considerations (managing counts of hydrogen bond donors, cLogP, molecular weight).^95^ Just like for PROTACs, the expanded PIP field would benefit from an expansion of small-molecule ligands that bind respective effectors with high potency and selectivity but do so without impairing their catalytic activity. One advantage of TPD over other PIP modalities is that many of the E3 ligases employed in TPD are substrate receptors of larger CRL assemblies and do not possess intrinsic enzymatic activity. This eliminates concerns about disrupting the catalytic function of these complexes, which may arise in modalities relying on the direct recruitment of proteins with catalytic activity, such as kinases or acetyltransferases. In cases where developing functionally “silent” ligands is not feasible, approaches such as allosteric binding site targeting, covalent ligand-directed release (CoLDR) chemistry, or cysteine-based group transfer chemistry offer innovative solutions.^96−98^ These methods provide avenues for overcoming challenges associated with maintaining target protein function while inducing proximity-based pharmacological effects, thus expanding the toolkit available for PIP-based strategies.

**Figure 4:**
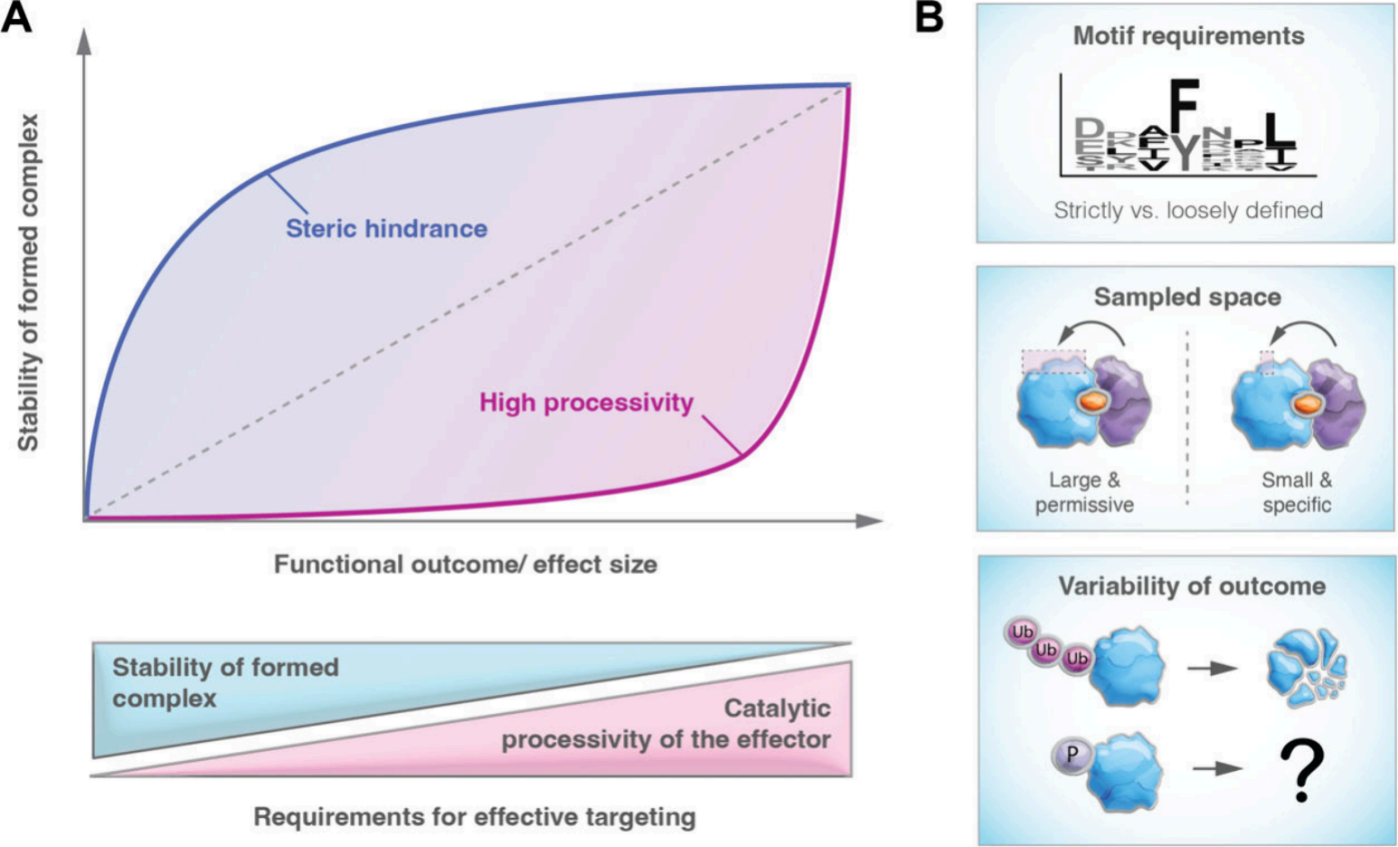
Unique characteristics of degrader-induced ternary complexes. (A) Schematic display of how effector processivity dictates the requirement for a stable ternary complex. (B) Other effector characteristics that influence success of proximity-inducing pharmacology. Schematically displayed are the requirements for a particular amino acid motif, the size of the sampled protein surface of the POI, as well as the variability of the outcome of a given posttranslational modification.

A central theme that emerged for degraders is the relevance of the ternary complex that is formed between the ligase, the degrader, and the target POI. Elegant studies utilizing SPR or ITC have demonstrated that the ternary complex binding affinity, stability, and cooperativity generally correlate with degradation potency.^99−101^ Here, cooperativity (α) represents the ratio of a degrader’s binary to ternary complex binding affinity. Positive cooperativity (α > 1) indicates that the tripartite assembly has a higher binding affinity than corresponding binary complexes. This often signifies that the underlying PPIs are favorable, while a negative cooperativity value (α<1) implies involvement of unfavorable PPIs. For TPD, it is crucial to remember that typically a chain of three ubiquitin moieties must be assembled on the POI to mark it for proteasomal degradation. This process often involves the interplay of a priming E3 ligase responsible for monoubiquitination and a second E3 ligase responsible for chain elongation, with monoubiquitination considered the rate-limiting step.^102^ This two-step mechanism may partially explain the significance of ternary complex stability in TPD. For other PIP modalities that depend on PTM alteration (each of which will come with their own rate and processivity), it will be important to understand if ternary complex kinetics will have a similar predictive power. For PIP approaches that lack a processive step but that are dependent on steric hindrance of the POI, stability of the ternary complex has an obvious, essential contribution to the compound activity. Examples are molecular glues such as rapamycin or cyclosporin that function by recruitment of FKBP12 or cyclophilin, respectively, molecular glues involving 14–3–3 proteins, or the recently introduced RIPTACs (Figure 4A).^103−107^

Another distinctive aspect that might separate TPD from other PIP modalities lies in the adaptability and plasticity of E3 ligases and the ubiquitin-proteasome system (UPS), which is evident across various layers. First, E3 ligases, particularly those belonging to the CRL family, typically possess a broad ubiquitination zone to accommodate substrates of diverse sizes and shape and can employ geometrically optimized catalytic partners for substrate targeting.^108,109^ This versatility means that multiple lysine residues on a potential substrate are likely accessible to the E3.^110,111^ Unlike certain functional effectors such as kinases, which often require specific motifs, most E3 ligases do not have such constraints. Second, K-48 poly ubiquitination can happen on various (different) lysine residues, all leading to the same, desired outcome of proteasomal degradation (assuming that chains of three or more ubiquitin moieties can be formed). Time will tell if other PIP systems are similarly permissive. For certain induced PTMs, such as phosphorylation, the relationship may be more complex. Phosphorylation of different residues could be functionally inconsequential or even lead to different outcomes. Consequently, there may be a greater need to ensure that a specific Ser/Thr/Tyr residue, which is *a priori* defined and functionally characterized, undergoes targeted phosphorylation or dephosphorylation. These nuances underscore the importance of understanding the intricacies of PTM induction in various PIP modalities and the potential challenges associated with achieving precise and predictable outcomes (Figure 4B).

**Figure 5:**
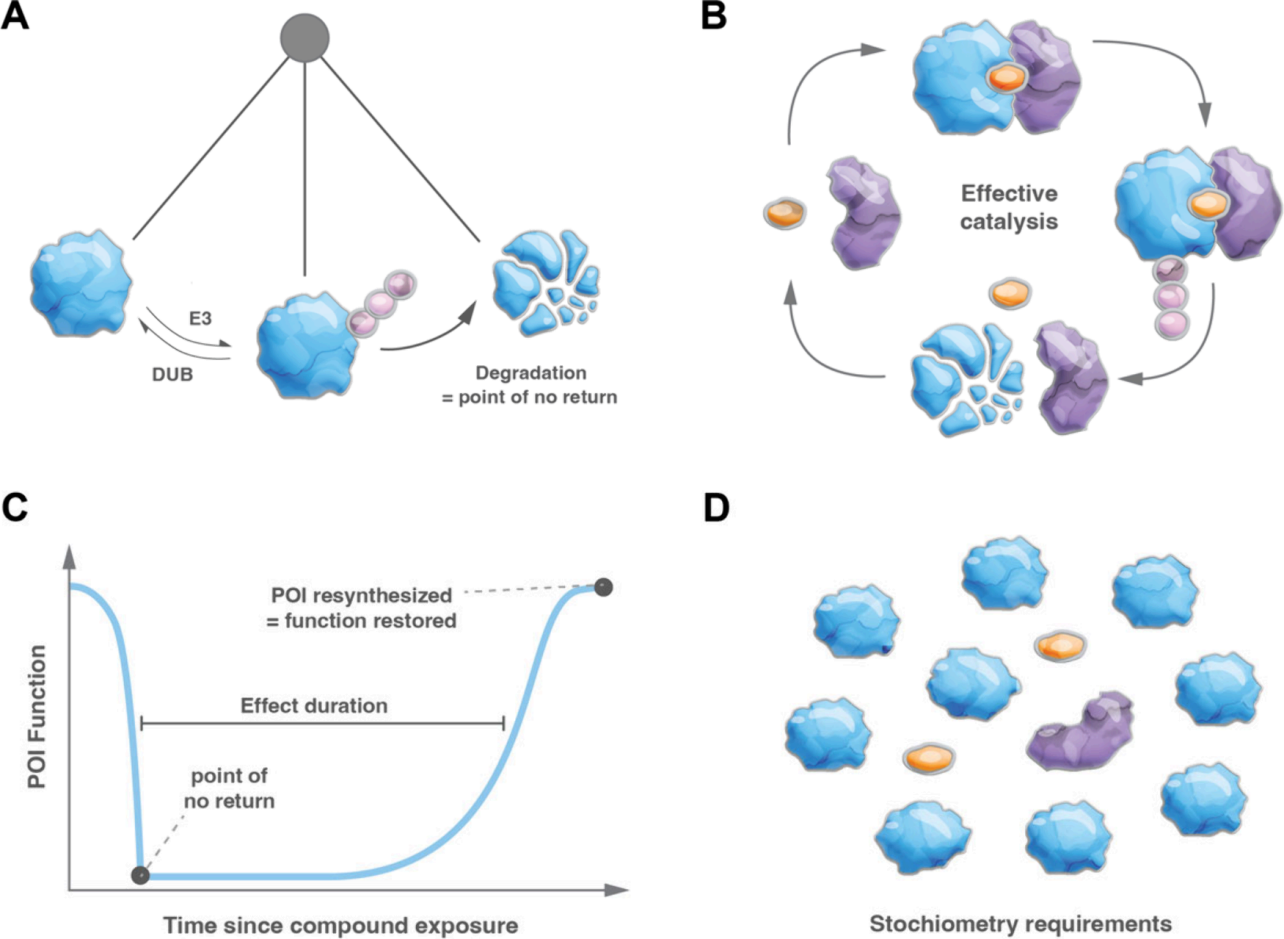
Relevance of the “point of no return” for the success of targeted protein degradation. (A) Schematic depiction of the interplay between E3/DUB in POI (de)ubiquitination and the point of no return that occurs when the poly ubiquitinated POI gets degraded by the proteasome. (B) The point of no return facilitates effective catalysis that is elicited by the degrader. This causes an extended duration of the functional effect (C) and puts less emphasis on favorable stoichiometry between POI and effector (D).

Third, it is arguable that every PIP-induced PTM is likely to be subject of an antagonistic enzymatic function. Acetylated lysines can undergo deacetylation, phosphorylated residues can be dephosphorylated, and ubiquitinated lysines can be deubiquitinated at different rates. Different PTMs are hence characterized by different kinetics. Changes in protein phosphorylation are, for instance, often evident already minutes after stimulation/perturbation.^112^ Alterations in acetylation typically manifest after several hours, while changes in methylation levels, for instance after blocking histone-lysine methyltransferases such as EZH2 or DOT1L, are frequently evident only days after perturbation.^113−115^ Based on the biological process that should be pharmacologically rewired, and based on the PTM that governs its regulation, even short-lived perturbations might however be sufficient (or even desired) to drive a biological response. Along these lines, it is crucial to note that the (de)ubiquitination cycle presents a critical and distinct “point of no return” once the substrate is degraded (Figure 5A). Notably, other PTM-inducing approaches might also offer similar points of no return. For example, when a PTM induces an allosteric change or a shift in intracellular localization that renders the protein less susceptible to PTM reversal. However, such mechanisms are likely not as widespread or as apparent as those involving the ubiquitination cycle. The point of no return also significantly contributes to the catalytic mode of action and prolonged effect duration of degraders. Degradation of the POI as the nonreversible end point facilitates catalysis and recycling of the degrader toward formation of another ternary complex, ubiquitination, and ensuing destruction. In contrast, if a catalytic small molecule induces a PTM that does not lead to POI elimination, its affinity for the posttranslationally modified target should be much lower than for the native form of the protein to avoid product-inhibited turnover (Figure 5B).^116^ Beyond facilitating the effective catalysis of the degrader, the point of no return enables the expanded duration of effect size that is frequently observed for degraders. This phenomenon describes the situation where a cell or tissue often exhibits attenuated protein levels for several hours after exposure to the degrader because the degrader-induced protein loss must be counteracted with compensatory protein resynthesis. Initially demonstrated with simple wash-out assays in tissue culture, this feature of degraders is one of the key distinguishing factors over conventional inhibitors in *in vivo* experiments and human clinical investigations (Figure 5C). It remains to be seen whether comparably extended effect durations will be observed for other PIP modalities. Lastly, and again due to the effective catalytic turnover that is prompted by degraders, the E3:POI stoichiometry is generally not considered to be an essential factor in effective degrader design and effective degradation has been observed for highly abundant proteins, such as FKBP12, which are expressed at much higher levels compared to the co-opted E3 (Figure 5D).^17,34,55,117^ Time will tell, if different effector stoichiometry will affect other proximity-inducing modalities.

Of note, not all PIP approaches depend on addition or removal of a defined PTM, yet they can still be catalytic. For instance, RIBOTACs function by inducing proximity between an RNA (via direct drug binding to structured RNA regions) and RNase L, thus inducing RNA degradation.^118,119^ Given that transcription is a process that is predominantly regulated at the level of proximity, it is maybe not surprising that proximity-inducing small molecules harbor the potential to reprogram transcriptional circuits, as initially shown by heterobifunctional ligands that recruit BRD4 to DNA in a sequence-specific manner, thus nucleating transcription elongation across repressive GAA repeats.^120^ More recently, transcriptional/epigenetic chemical inducers of proximity (TCIPs) were described. TCIPs are heterobifunctional ligands that recruit transcriptional activators (such as BRD4 or the estrogen receptor) to the transcriptional repressor BCL6, thus actively derepressing (activating) BCL6-bound loci, such as pro-apoptotic genes.^121^ In my opinion, this is one of the most promising future research avenues with many fundamentally important learnings in front of us. For instance, it will be crucial to understand the significance of the relative abundance between the two recruited factors and to functionally identify markers for genomic regions that are permissive to small-molecule reprogramming. Additionally, identifying ideal complementary effectors to pharmacologically activate or repress these regions will be essential (see ensuing section).^122−124^ From a chemistry perspective, rewiring transcription regulation will require ligand discovery efforts for transcription factors or transcriptional activators, such as the Mediator complex. Given that many of these effectors are devoid of deep hydrophobic pockets, I foresee a prominent role in covalent chemistry approaches in these ventures.^5^ To the best of my knowledge, most PIP modalities are thus far reliant on heterobifunctional ligands. To broaden the scope, target space and opportunities, I additionally anticipate an expansion toward monovalent, glue-like compounds. It seems reasonable to assume that the growing set of rules for MGDs will similarly apply. For instance, identifying monovalent PIP modalities may be easier when trying to stabilize interactions between proteins that already have some baseline affinity for each other.^15,125^ Interestingly, there is proof-of-concept for molecular glues that stabilize RNA:protein interactions to prompt a functional cellular consequence. Risdiplam is an FDA-approved drug for the treatment of spinal muscular atrophy that was initially identified via a phenotypic screen.^126^ Mechanistically, it functions by stabilizing the interaction between the 5′ splice site of exon 7 of the *SMN* gene and the U1 small nuclear ribonucleoprotein (snRNP) of the spliceosome, thus leading to the preferential inclusion of exon 7 and an increase in functional SMN protein levels.^127^ Additionally, recent work has identified an electrophilic ligand that stabilizes interactions between RNA and the RNA-binding protein NONO, thus suppressing transcription of the androgen receptor.^128^ Further research in these non-PTM inducing strategies will be required to develop approaches to rationally identify or design compounds with glue-like features.

## What’s Next and Where Are We Going?

In summary, the long-term goal of chemical neomorphs is to enable synthetic biology without the need for labor intensive (and therapeutically often impractical or infeasible) genetic engineering. The goal is to install, with all pharmacologic advantages such as dose dependency or reversibility, biological circuits that operate beyond evolutionary constraints. This task is certainly as daunting as it is exciting, and we will both learn the possibilities as well as the limitations of the achievable complexity of the circuitry that can be reprogrammed by small molecules. But with an almost infinite chemical space and ever-increasing possibilities in high-throughput and pooled synthetic strategies, I am optimistic that in a decade from now we will be able to fashion chemical neomorphs that will enable us to generate more complex functional circuits than it is feasible today. I would predict that chemical induction of proximity will remain the main conceptual workhorse in these attempts. This puts a spotlight on our ability to better understand which compound-induced PPIs are functional, and how likely two proteins can be connected/glued together. To map inducible, functional PPIs in mammalian cells, several high-throughput approaches have been reported, allowing for instance to identify transcriptional activators and repressors, as well as effectors of protein degradation and stabilization.^122−124,129^ To map the compatibility of two proteins for induced proximity applications, advances in predicting protein structures and higher-order protein assemblies are expected to play an important role.^130,131^ In addition, genetic variation can point to inducible and functional PPIs. Arrayed experiments that mapped interactomes of wild-type proteins and known cancer-driving mutants have for instance revealed many variant-enabled neomorphic protein–protein interactions (neoPPIs).^132^ Further, deep mutational scanning (DMS) experiments allow to systematically vary individual protein domains or entire proteins in a pooled format, and to assess how genetic variation affects a predefined readout. DMS experiments with CRBN previously allowed us to identify variations that augment the activity of assayed CDK9 PROTACs, using a positive selection assay that is based on cellular fitness.^37^ This finding is in line with the fact that the involved protein surfaces (CRBN:CDK9) are not evolutionary optimized to be connected, which is expected for a bifunctional molecule. Future experimental setups that rely on selectable binding assays (rather than a readout based on cellular fitness) could inform if genetic adaptation of a protein interface can stabilize interactions with another effector protein. Noteworthy, comparable experimental setups (based on protein complementation assays) have already been successfully utilized in yeast to map allosteric sites in proteins.^133,134^ Future research will be required to understand if such genetically identified, tunable regions can be harnessed for the discovery of ligands that would then chemically mimic the genetic surface adaptation and stabilize a PPI of interest. In addition to structure-based drug discovery approaches, DNA encoded library-based selections and chemoproteomics screens could serve as valuable tools to reach this ambitious goal.^135−137^ Of course, many of the aforementioned principles will, with technical adaptations, similarly apply to identify chemical approaches to stabilize protein-RNA or protein–DNA interactions. To that end, our field will undoubtably benefit from a deeper understanding of how small molecules can interact with RNA, either in its free form or when complexed with proteins, where cryptic pockets might reveal itself.^138^ As we are learning more about proximity-inducing strategies, we will learn which modalities best match clinical requirements for a particular target and a given disease setup. The path to the clinic for degraders has, until now, predominantly led to oncology. However, promising initial clinical data for IRAK4 degraders in inflammatory diseases, such as atopic dermatitis, suggest that TPD should have broad applicability and reach in other indications.^139^ For neomorphic approaches beyond TPD, similar considerations will have to be made. Given that some of these approaches allow for more subtle (and presumably less durable) interventions, their first clinical indications might be outside of oncology.

In conclusion, empowered by insights and technologies that were inconceivable only a decade ago, our creativity appears to emerge as the rate-limiting factor in our survey for chemical neomorphs that can reprogram cellular circuitry.
